# Targeted metabolomics unravels the mechanism by phenylpropanoid-rich of the peel of *Zea mays* L. ameliorates metabolic disorders in diabetic mice through gut microbiota modulation

**DOI:** 10.3389/fphar.2025.1551713

**Published:** 2025-04-09

**Authors:** Xiaotian Cheng, Jinyan He, Yuru Yang, Yaonan He, Guangtong Chen, Bai Ling, Andong Wang

**Affiliations:** ^1^ School of Pharmacy, Nantong University, Nantong, Jiangsu, China; ^2^ Department of Pharmacy, The Yancheng Clinical College of Xuzhou Medical University & The First people’s Hospital of Yancheng, Yancheng, Jiangsu, China

**Keywords:** metabolic disorders, *Zea mays* L., gut microbiome, metabonomics, diabetes

## Abstract

**Background:**

Diabetes is one common clinical symptoms of metabolic disorders. The peel of *Zea mays* L. is a folk remedy for diabetes that has not been thoroughly studied. The effects and mechanisms on diabetes complicated glucose and lipid metabolism disorders are still unknown now.

**Purpose:**

The research is intended to elucidate the constituent of phenylpropanoid enriched of *Zea mays* L. (YMP), and investigate the treatment and mechanism on amending glucose and lipid metabolism disorders.

**Methods:**

The constituents of YMP were systematacially identified by HPLC-Q-TOF-MS/MS and NMR. To assess the effects of varying YMP doses, diabetic mice induced by streptozotocin and a high-fat diet were divided into groups. Targeted serum metabolomics investigations were conducted using UHPLC-LTQ-Orbitrap MS. Moreover, 16S rRNA analysis was employed to elucidate the intricate mechanisms through the gut microbiota modulates lipid and glucose metabolism.

**Results:**

It demonstrated that the primary component of YMP was luteolin. At a high dosage of 160 mg/kg/day, YMP considerably reduced the values of the oral glucose tolerance test, insulin, and blood glucose (*p* < 0.001). After administration, insulin resistance indexes decreased. YMP reversed the accumulation of glycogen in the liver and reduced hepatic lipid deposition. Compared to MOD group, the concentration of luteolin is higher and its metabolite, indicating that luteolin may be adequately absorbed and have an influence on the circulatory system. The results of 16S rRNA sequencing demonstrated that YMP and gut microbiota interacted to positively regulate beneficial bacteria such as *Bifidobacterium*, *Ligilactobacillus,* and *Lactobacillus*.

**Conclusion:**

This work investigated the regulating effect of YMP on the liver glycolipid metabolism for the first time, and it also showed the underlying mechanism through gut microbiota. According to these studies, YMP has a lot of potential to be used as a supplemental treatment for complex metabolic illnesses like diabetes. It offered empirical support for the use of alternative medicine in the area to treat complex problems of glucose and lipid metabolism in diabetes.

## Introduction

A combination of metabolic illnesses together with related conditions, including dyslipidemia, hyperglycemia, hypertension, central adiposity, insulin resistance, and so on, is known as metabolic disorder, a global health issue (Fahed et al., 2022). Among them, diabetes is the most common clinical manifestation, along with complex abnormalities of glucose and lipid metabolism disorders (Poznyak et al., 2020). Type II diabetes mellitus (T2DM) occurrring during the progression of metabolic disorder is a condition marked by inflammation, insulin resistance, abdominal obesity, and high blood sugar, which often accompanied by high blood pressure. Increasing papers showed that diabetes-related metabolic disorders were complicated, not just keeping blood sugar levels at reasonable ranges for its therapeutic purpose. Therefore, an exhaustive plan for addressing metabolic disorders should be proposed. Natural products, for example, are supplementary therapies for diseases due to their safety, multitargets and multiple biological effects (Guo et al., 2022).

*Zea* is one of the most significant food crops in the Gramineae family, which has about 10,000 species. The material that makes up *Zea* husk was brought to China during the Ming Dynasty (Zhang et al., 2023). It is thought to be a diuretic that can treat edema, urinary stones, unpleasant urination, and appetite loss. Acute and chronic nephritis, edema, proteinuria, and diabetes are among the conditions for which *Zea* is listed as a medicine in the “Dian nan Materia Medica”, “Ling nan Caiyao Lu”, “Modern Practical Chinese Medicine”, “Dictionary of Traditional Chinese Medicine”, and numerous other Chinese medical texts. *Zea* husks received less attention in earlier research than *Zea* silk (Bispo et al., 2021).

Dietary polyphenols, which are abundant in a variety of fruits, cereals, and vegetables, are used as phytotherapies for long-term illnesses (Goya and de Pascual-Teresa, 2023). Dietary polyphenols have the ability to effectively lower the risks associated with cardiometabolism and are distinguished by their hydroxylated phenyl moieties, substitution groups, and phenolic rings. After an 8-week polyphenols-diet, serum zonulin levels decreased. They also increased considerably from 812 mg/day to 1,391 mg/day, showing a significant effect of treatment (*p* < 0.01) and treatment × time interaction (*p* < 0.01). Furthermore, a decrease in diastolic blood pressure (*p* < 0.01) was observed in the treatment × time interaction results after the polyphenols-diet, with this effect being greatest in individuals who were not using antihypertensive medications. Women showed a drop in both diastolic (*p* < 0.01) and systolic (*p* < 0.01) blood pressure (Del Bo et al., 2021). After consuming food or liquids, dietary polyphenols affect the glucose transporters absorb glucose of small intestine by interacting with them. For instance, the apple polyphenol phlorizin inhibits sodium-glucose linked transporter-1. The brush border digestive enzyme lactase then converts phlorizin to phloretin, a potent inhibitor of glucose transporter-2 (GLUT2), in the intestinal lumen. Thus, after a glucose challenge, phlorizin-rich apple extract lowers insulin and blood glucose in healthy participants. Conversely, oleuropein, an olive phenolic, inhibits GLUT2, although not to the extent necessary to modify blood glucose in healthy individuals following a glucose challenge (Williamson, 2022). Epigallocatechin-3-gallate (EGCG) prophylaxis reduced colitis and markedly increased the abundance of bacteria that produce short-chain fatty acids (SCFAs), such *Akkermansia*. In comparison to the microbiota from CON group, the microbiota from EGCG-dosed mice improved colonic barrier integrity, reduced colitis, and enhanced bacteria, especially *Akkermansia*. Collectively, the reduction of colitis caused by oral EGCG points to a close relationship with SCFA-producing bacteria, particularly *Akkermansia* (Wu et al., 2021).

Few research has examined the holistic chemical profile or quality evaluation related to the traditional effects of YMP, despite the fact that multi-constituent determination methodologies have been used for YMP quality evaluation. In general, it is still unknown about the identified chemicals related to the therapeutic benefits. The aim of this study was investigated at the components of YMP and explored therapy options for diabetes *in vivo*, including dyslipidemia, hepatic steatosis, insulin resistance, and hyperglycemia. Furthermore, to identify the potential mechanisms of YMP treatment, the gut microbiota and metabolomics were investigated.

## Materials and methods

### Extraction of YMP and major constituent

The YMP was collected and extracted three times (2 h each time) using 95% EtOH in a reflux environment. To obtain the 95% ethanolic extracts, the solvent was evaporated using a vacuum rotary evaporator. The S-8 macroporous resin was used to separate the ethanol extract. S-8 macroporous resin was first eluted using a three-fold bed volume of water, and it was subsequently eluted and collected at a 2 mL/min flow rate using a three-fold bed volume of 30% ethanol. YMP was purified by semipreparative HPLC using a YMG C18 (20 × 250 mm) column and gradient elution mobile phase acetonitrile at a flow rate of 2 mL/min for 0 min (20%), 5 min (30%), 30 min (60%), and 55 min (90%). The major compound was identified according to standard methods. Purified sample luteolin was dissolved in 0.5 mL DMSO. ^1^H-NMR and ^13^C-NMR spectra were recorded on a 600 MHz spectrometer (Bruker, Germany).

### Establishment of animal model

A total of fifty (20 ± 2 g) mice were acquired from Beijing HFK Bioscience Co., Ltd (Beijing, China) and kept in cages with free access to food and water, 12 h of light and dark cycles, and 25°C ± 1 °C and 55% ± 5% relative humidity. The Nantong University Animal Ethical and Welfare Committee examined and approved the animal protocol (R240411744). The animal ethical and welfare was approved on April 25th in 2024 with an Approve No. S20240425-006.

Following a week of acclimation, 10 mice were chosen at random to be the control group (CON), which had a normal diet, while the other mice received a 45% high-fat diet (HFD). Each treatment group also had 10 mice. From the beginning of the experiment, two YMP-treated groups (40 and 160 mg/kg/d; called the YMPL and YMPH groups, respectively) were given YMP injections into HFD mice. The dosage was determined by our previous pre-experiment. Following a 4-week period, the HFD mice underwent a 12-h fast followed by intraperitoneal injections of streptozotocin (50 mg/kg/d, prechilled 0.1 M/L sodium citrate buffer, pH 4.2) for four consecutive days, while the CON mice received sodium citrate buffer injections. After 3 days of STZ treatment, two groups were randomly selected: the luteolin group (160 mg/kg/d, LUT) and the model group (MOD). The dosages were chosen with consideration for safety, drawing from earlier research. Blood glucose levels and changes in body weight were also routinely documented. Following therapy, the animals were slaughtered in accordance with animal welfare laws, and mouse blood was taken for biochemical indicator analysis.

### Serum biochemical analysis

Low-density lipoprotein cholesterol (LDL-C), high-density lipoprotein cholesterol (HDL-C), total cholesterol (TC), triglycerides (TG), were measured in the serum using commercial assay kits (Nanjing Jiancheng Technology Co., Ltd.). Elisa assay kits (Elabscience Biotechnology Co., Ltd.) were used to measure the elevation of blood fasting insulin level (FIN).

### Insulin resistance and oral glucose tolerance test

The oral glucose tolerance test (OGTT) was administered using accepted practices. In short, mice were given an oral glucose dose of 2 g/kg 2 days before to administering YMP after an overnight fast. After the glucose was administered, blood glucose levels were measured at 0, 30, 60, 90, 120, and 180 min. AUC over 180 min was used to compute the OGTT. The following formula was used to determine three main indexes:

$$\text{HOMA}-\text{IR}=\text{FBG}\times \text{FIN}/22.5;\text{ HOMA}-\beta =\left(20\times \text{FIN}\right)/\left(\text{FBG}-3.5\right);\text{ QUICKI}=1/\left(\mathrm{lg}\text{FBG}+\mathrm{lg}\text{FIN}\right).$$

### Histopathological analysis

For additional staining, the samples were preserved in a 10% formaldehyde solution. Oil red O was used to stain the frozen sections, and hematoxylin-eosin (H&E) and periodic acid Schiff (PAS) staining were applied to the paraffin sections. A Leica DM 1 inverted microscope was used to view and take pictures of the liver tissues.

### Sample preparation for metabolomics analysis

The serum sample was mixed with three times. The sample was centrifuged for 20 min at 10,000 rpm (4°C) after being combined for 3 min. To conduct a focused metabolomics investigation, the supernatant was removed. A centrifuge tube was filled with 100 µL serum, 190 µL methanol, 380 µL dichloromethane, and 120 µL water. The tube was vortexed for 3 min, and then centrifuged for 15 min at 12,000 rpm (4°C). 220 μL of acetonitrile-water (v/v, 4/1) was used to resuspend the 250 µL of supernatant that had evaporated under nitrogen gas. Following centrifugation, 10 µL of IS solution (synephrine in positive mode) was added to 200 µL of sample. For a targeted metabolomics research, the material was centrifuged for 10 min at 10,000 rpm (4°C). Samples for quality control (QC): By combining 10 μL of each serum sample and centrifuging the mixture, serum quality control samples were produced.

### Phenylpropanoid-targeted metabolomics analysis conditions

A metabolomics investigation focused on phenylpropanoid targeting was conducted using the Ultimate 3000 UHPLC system. To separate the samples, a Waters UPLC HSS T3 column (1.8 μm, 2.1 mm × 100 mm) was used. Acetonitrile served as mobile phase B and water with 0.1% (v/v) formic acid as mobile phase A. 0.3 mL/min is the flow rate. The following gradient elution program was used: 5% B for 0–2 min, 5%–60% B for 2–5 min, 60%–95% B for 5–12 min, 95% B for 12–20 min, 95%–5% B for 20–20.1 min, and 5% B for 20.1–25 min. Every seven samples in the analysis queue, a quality control sample was added. Volume of injection: 8 µL. 35°C is the column temperature. For testing, LTQ Orbitrap Velos Pro was employed. A source of electrospray ionization (ESI) was employed. This work was performed based on positive and negative ion modes.

### Metabolomics analysis

Unsupervised principal component analysis (PCA) and orthogonal partial least squares discriminant analysis (OPLS-DA) were the main methods used for multivariate statistical analyses. The load plot S-plot and variable importance in projection (VIP) plot were utilized to illustrate the degree of metabolite contribution during the OPLS-DA analysis. It might quantify the degree to which each metabolite influences how samples from each group are categorized according to the VIP value. The t-test was used to further confirm if there was a significant difference between the groups. Differential metabolites were defined as those with VIP >1, t-test (p < 0.05), and fold change (FC) values (FC > 1.5 or <0.7).

### SCFAs analysis

With some changes, the short chain fatty acids (SCFAs) in 100 mg of stool samples were calculated using a prior study’s methodology. Diethyl ether (500 μL), 10% H_2_SO_4_ (160 μL), and 50 μL of dH_2_O were used to sequence the extraction system. Gas chromatography in conjunction with a flame ionization detector (Agilent 7890B, United States) outfitted with a DB-WAX column (30 m × 0.32 mm × 0.25 μm, Agilent 123–7032) was used to examine SCFAs.

### Gut microbiota analysis

As per earlier procedures, fresh feces samples were gathered in liquid nitrogen for high-throughput sequencing analysis. For the beta diversity analysis, feature-level alpha diversity indices were computed using principal coordinate analysis (PCoA) and cluster analysis, using observed OTUs, the Chao1 richness estimation, and the Shannon diversity index. The significances between different groups were examined using the LEfSe program. To ascertain the connection between the gut microbiota and host metabolism markers, Spearman’s correlations were utilized.

### Statistical analysis

The program SPSS 22.0 was used to evaluate the data. The mean ± standard deviation was used to illustrate the results. One-way ANOVA and the Tukey post-test were used to generate group comparisons, with p < 0.05 considered statistically significant.

## Results and discussion

### Isolation and compounds identification of YMP

**FIGURE 1:**
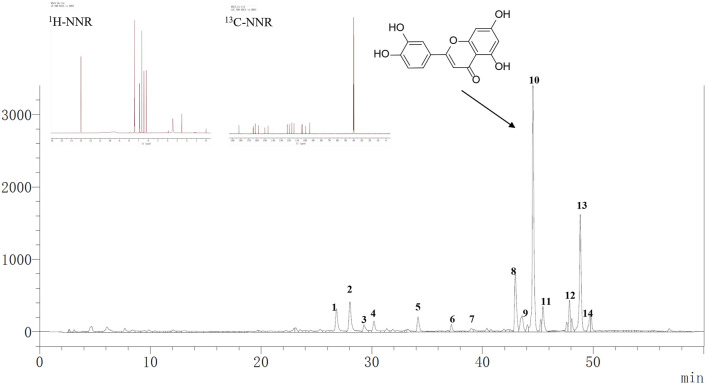
The chromatogram of YMP, and ^1^H-NMR, ^13^C-NMR of Luteolin.

S-8 macroporous resin is a polar copolymer polymerized from styrene. Following S-8 treatment, the YMP yield was around 39.02%. With a substantial enrichment of 67.51 percent (*p* < 0.001), the content of total polyphenols in SAP was increased from 0.32 ± 0.02 to 0.53 ± 0.03 mg gallic acid equivalent per milligram of dry crude ethanol extract. S-8 macroporous resin proved to be a useful substance for increasing overall polyphenol content. The major fraction 10 displayed the ^1^H-NMR and ^13^C-NMR spectra. Following examination and comparison with earlier research, it was determined to be highly pure luteolin. By using HPLC-Q-TOF-MS/MS, the compounds of YMP were tentatively identified. Figure 1 displayed the base peak chromatogram, and Table 1 contained a list of the probable compounds. The literature and the PubChem database (https://pubchem.ncbi.nlm.nih.gov/) were used to identify the chemicals. YMP was primarily composed of luteolin.

**TABLE 1:** The tentative compounds identification of YMP.

Peak	Rt (min)	Compound identified	Molecular formula	Theoretical Mass (*m/z*)	Accurated Mass (*m/z)*	MS/MS	Error (ppm)	Ref
1	26.8	Chlorogenic acid	C_16_H_18_O_9_	354.0954	354.0951	163,355	0.99	Bai et al. (2023)
2	28	Genistin	C_21_H_20_O_10_	432.1059	432.1056	239,268	0.49	Farooq et al. (2020)
3	29.3	Homovanillyl alcohol 4-O-glucoside	C_15_H_22_O_8_	330.1319	330.1315	163,255	1.2	Domínguez-Perles et al. (2017)
4	30.2	Querciturone	C_21_H_18_O_13_	478.0747	478.0747	151,301	−0.17	Lin et al. (2022)
5	34.2	Luteolin-7-O-glucuronside	C_21_H_18_O_12_	462.0785	462.0798	153,287,307	−2.94	Seger and Salzmann (2020)
6	37.2	Quercitrin	C_21_H_20_O_11_	448.1008	448.1006	303,345,369,413	0.43	Yu et al. (2018)
7	39	Quercetin	C_15_H_10_O_7_	302.0417	302.0427	165,257,285	−3.3	Lin et al. (2022)
8	43	Isolicoflavonol	C_20_H_18_O_6_	354.1103	354.1103	146,189	0.01	Wang et al. (2014)
9	43.6	Cianidanol	C_15_H_14_O_6_	290.0792	290.079	163,241	0.54	Abdulhafiz et al. (2022)
10	44.6	Luteolin	C_15_H_10_O_6_	286.0473	286.0477	244,289	−1.52	Wu et al. (2022)
11	45.5	Lupiwighteone	C_20_H_18_O_5_	338.1148	338.1154	165,357	−1.91	Kobayashi and Imai (2021)
12	47.9	5-Hydroxy-3,6,7,8,3′,4′-hexamethoxyflavone	C_21_H_22_O_9_	418.1258	418.1264	328,346,361	−1.32	Li et al. (2023)
13	48.8	Chrysosplenetin	C_19_H_18_O_8_	374.1005	374.1002	237,266	0.86	Wang et al. (2023)
14	49.7	Cirsimaritin	C_17_H_14_O_6_	315.086	314.079	136,226	1.12	Ren et al. (2020)

### Hypoglycemic analysis

**FIGURE 2:**
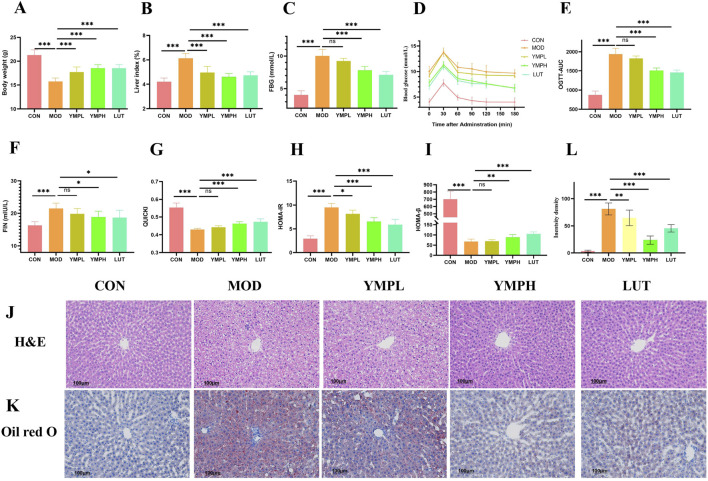
The effects of YMP on blood glucose related indexes, including body weight gain **(A)**, liver index **(B)**, fast blood glucose **(C)**, oral glucose tolerance test **(D)**, OGTT **(E)**, FIN **(F)**, QUICKI **(G)**, HOMA-IR **(H)**, HOMA-β **(I)**, H&E staining **(J)**, Oil red O staining **(K)**, Relative density of Oil red O staining **(L)** (×200) Note: ^*^ represents for vs. MOD group (*p* < 0.05), ^**^ represents for vs. MOD group (*p* < 0.01), ^***^ represents for vs. MOD group (*p* < 0.001), respectively. N = 8.

The mice displayed diabetes signs, such as being skinny, polydipsia, and having polyuria, after receiving therapy with STZ and HFD, suggesting the availability of diabetic models. When compared to the MOD group, the CON groups showed a notable increase in body weight. The diabetic mice changes much; nevertheless, the group receiving a high dosage of YMP and LUT groups exhibited evidence of maintaining their weight (Figure 2A). It is worth noting that the organ index of diabetic mice was substantially higher (*p* < 0.001) than that of the CON group, suggesting that the organ index could be used to identify the toxicity of medications. A statistically significant difference was seen between YMPH and the other groups, indicating a protective effect against STZ damage (*p* < 0.001) (Figure 2B). YMP at 160 mg/kg might considerably lower the FBG in comparison to the model group (*p* < 0.001) (Figure 2C).

### Glucose tolerance analysis

After intragastric glucose injection, OGTT exhibits a rapid rise in blood glucose within 30 min and a gradual return to normalcy after 90 min (Figures 2D, E). After 180 min, we discovered that the mice were unable to regulate their blood glucose levels and that the MOD group’s blood glucose concentration was still high. The YMPH and LUT groups exhibited a considerably lower area under the curve (AUC) (*p* < 0.001) in comparison to the MOD group, indicating their good glucose tolerance. Regretfully, YMPL did not exhibit a significant enough downregulation of glucose (*p* > 0.05). Based on these findings, we concluded that controlling T2DM and halting the degradation of required substantial absolute doses of different polyphenols.

### Insulin related analysis

Insulin resistance was observed in mice fed a high-fat diet for several weeks, during which time their tissue exhibited a passive response to insulin and reduced glucose absorption efficiency, resulting in compensatory increased insulin release (Figure 2F). The insulin resistance characteristics of type II diabetes are suggested by the lower QUICKI and HOMA-β indexes as well as the higher blood HOMA-IR indexes in diabetic mice (Figures 2G–I). The QUICKI value is a useful tool for assessing how sensitive target organs are to insulin. Research has demonstrated that YMP concentrations can upregulate QUICKI and enhance organ sensitivity to insulin. While the YMP treatment group did not have a statistically significant decrease in insulin levels, there was a similar small downregulation trend that was concentration-dependent. The HOMA-IR score showed a significant declining trend (*p* < 0.001) at high concentration. When comparing the diabetic mice group to the CON group, the HOMA-β value was significantly lower, indicating significant damage to the pancreatic islet β-cells and disruption of the insulin secretion function (*p* < 0.001).

### H&E staining

Histological examination showed that YMP reduced the lipid build-up and severe pathological alterations caused by HFD and STZ, including localized necrosis, vacuolar degeneration, mussy hepatic cords, and congestion in the central vein (CCV), along with lymphocyte infiltration in the MOD group. On the other hand, animals administered YMP exhibited comparatively regular organization and CCV, which aligned with the liver function results (Figure 2J). As anticipated, the hepatic tissues stained with oil red O in the diabetic group clearly displayed lipid buildup. Treatment with YMP markedly improved the lipid droplets and vacuoles (*p* < 0.001, Figures 2K, L). The function of YMP on hepatic dyslipidemia was confirmed by the oil red O findings in the liver tissue section. Similarly, supplementing STZ-induced diabetic mice with a polyphenol-rich extract improved their fasting blood glucose and reduced dyslipidemia, adding to the data supporting the use of natural polyphenol.

### Lipid metabolism analysis

**FIGURE 3:**
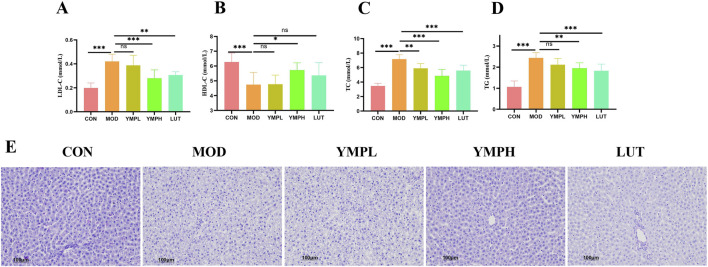
The effects of YMP on lipid metabolism, including LDL-C **(A)**, HDL-C **(B)**, TC **(C)**, TG **(D)**, PAS staining **(E)** (×200) Note: ^*^ represents for vs. MOD group (*p* < 0.05), ^**^ represents for vs. MOD group (*p* < 0.01), ^***^ represents for vs. MOD group (*p* < 0.001), respectively. n = 8.

In addition to T2DM, HFD increases the risk of dyslipidemia, which in turn causes non-alcoholic fatty liver disease (NAFLD). The dyslipidemia biochemical indicators (LDL-C, HDL-C, TC, and TG) in serum were assessed in order to determine the lipid build-up and development of steatosis in the liver (Figures 3A–D). These showed that following YMP delivery, TC, TG, and LDL-C were much lower in comparison to diabetic mice, whereas the level of HDL-C exhibited a considerable increase following high YMP consumption. In particular, LDL-C level of YMPH was reversed as compared to the MOD group, while most lipid metabolic indicators showed little to no change at low dosages. The amount of glycogen in the liver was measured using PAS staining, which is seen in the purplish red hue. The MOD group exhibited ballooning degeneration with nuclear vacuolization of hepatocytes in the liver slice (Figure 3E). Decreased glycogen deposition causes the synthesis of fat to be diverted from glucose, which worsens glucose metabolism and increases insulin production to bring serum glucose levels back to normal (Oluranti et al., 2021). As previously shown, YMP dramatically reduced insulin resistance, which increased the amount of glycogen deposited in the liver and had a favorable impact on blood glucose and fat storage. YMP showed benefits in treating diabetes combined with liver metabolic problems based on the control of liver function and glycolipid metabolism.

### Phenylpropanoid-targeted metabolomics analysis

Targeted metabolomics can understand the metabolic pathways, metabolites and their mechanisms of action of drugs in the human body, thereby optimizing the design and development process of drugs. Targeted metabolomics analysis was used to investigate the metabolism of YMP, and serum samples underwent positive ionization modalities. PCA was an unsupervised technique for analyzing data that reduced dimensionality by using an orthogonal transformation. The serum samples were clearly separated from the mice’s normal counterparts. The result indicated model mice had abnormal metabolism. In positive modes, 151 metabolites were found in serum samples.

**FIGURE 4:**
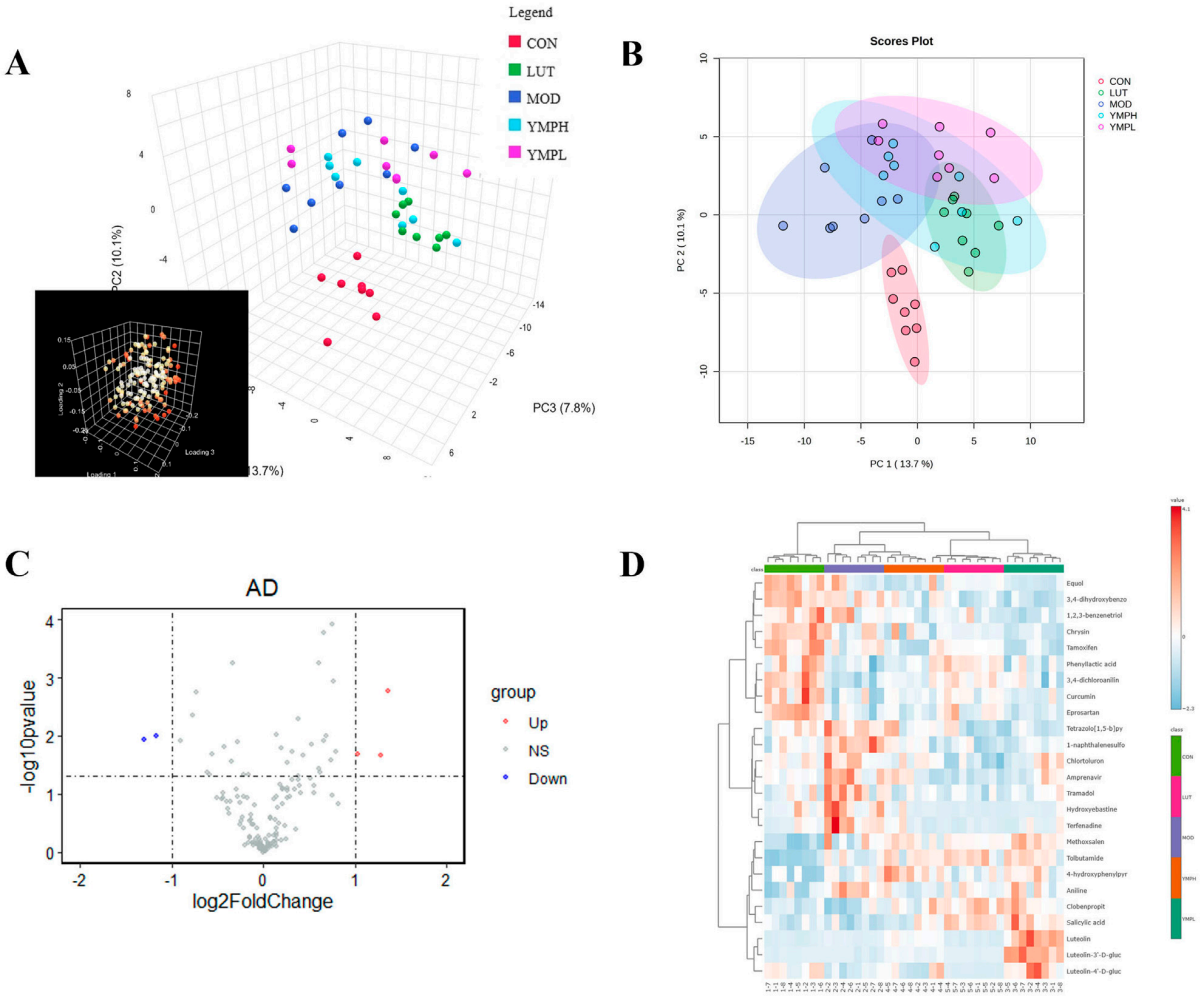
Metabolite changes following the YMP intervention in positive mode. The picture of PCA 3D plots **(A)**, the picture of PCA 2D plots **(B)**, the volcano plot of serum samples between MOD and YMPH **(C)**, Top 25 thermodynamic diagram **(D)**.

Based on the global features of the raw data, PCA (Figures 4A, B) and OPLS-DA analysis were carried out to distinguish between the groups and visualize the metabolic differences. Furthermore, five groups showed a considerable distinction as shown by PCA score plots. There were clear differences between the various metabolite categories based on variations in physiological markers. OPLS-DA might eliminate negligible differences while also increasing classification accuracy (Supplementary Figures S1A, C, E, G). The two groups could be distinguished from one another using the OPLS-DA model score plots. In MOD groups, endogenous chemical metabolisms were changed. Biomarkers could be identified using metabolic profiles. In serum samples, the parameters of R2Y and Q2 were 0.991 and 0.839 (CON vs. MOD), 0.977 and 0.853 (MOD vs. YMPL), 0.977 and 0.704 (MOD vs. YMPH), 0.987 and 0.891 (MOD vs. LUT) in positive ion mode (Supplementary Figures S1B, D, F, H). The OPLS-DA score plots of model mice were obviously distinguished from MOD groups. The concentration of luteolin (Log_2_ FC = 1.44 and -Log_10_ FDR = 2.79) is higher than MOD groups, which mean luteolin could be sufficiently absorbed and showed effect by blood system (Figure 4C). At the same time, it was observed that the metabolites of luteolin (Luteolin 7-glucuronide, Luteolin-3′-D-glucuronide, Luteolin-4′-D-glucuronide) were also higher than MOD groups (Figure 4D).

### YMP consumption changed microbiome composition and microecology

Numerous studies have shown that polyphenols affect the gut microbiota and change the composition and dynamics of communities. For the first time, the reciprocal relationships between YMP and gut microbiota were verified using gut microbiome. After gathering the fresh feces, 16S rRNA sequencing was performed on a Novogene platform. A total of 1,794,180 sequences of excellent quality were gathered, with an average sequence number of 89,709 ± 8,765 for every sample. 3,956 OTUs were formed from the effective sequences at a 97% similarity level. The logic and efficiency of the sequencing depth were shown by the rarefaction curve approaching the plateau, and the plateaued Shannon indices showed that there was enough sequencing data to describe the variety in the current sample (Supplementary Figures S2A, B).

Through the diversity of individual samples, alpha diversity represents the diversity and richness of microbial communities. A low alpha diversity is thought to be linked to a higher risk of long-term metabolic diseases. In addition to the Chao1 level of YMPH being piratically higher than MOD groups, the community richness between CON and MOD groups differed significantly (Supplementary Figure S2C). The Shannon and Simpson index levels of YMPH were also significantly greater than those of MOD groups, indicating a minor alteration of community diversity. Lower levels of these indicators were seen in the MOD groups compared to YMP groups (Supplementary Figures S2D, E).

**FIGURE 5:**
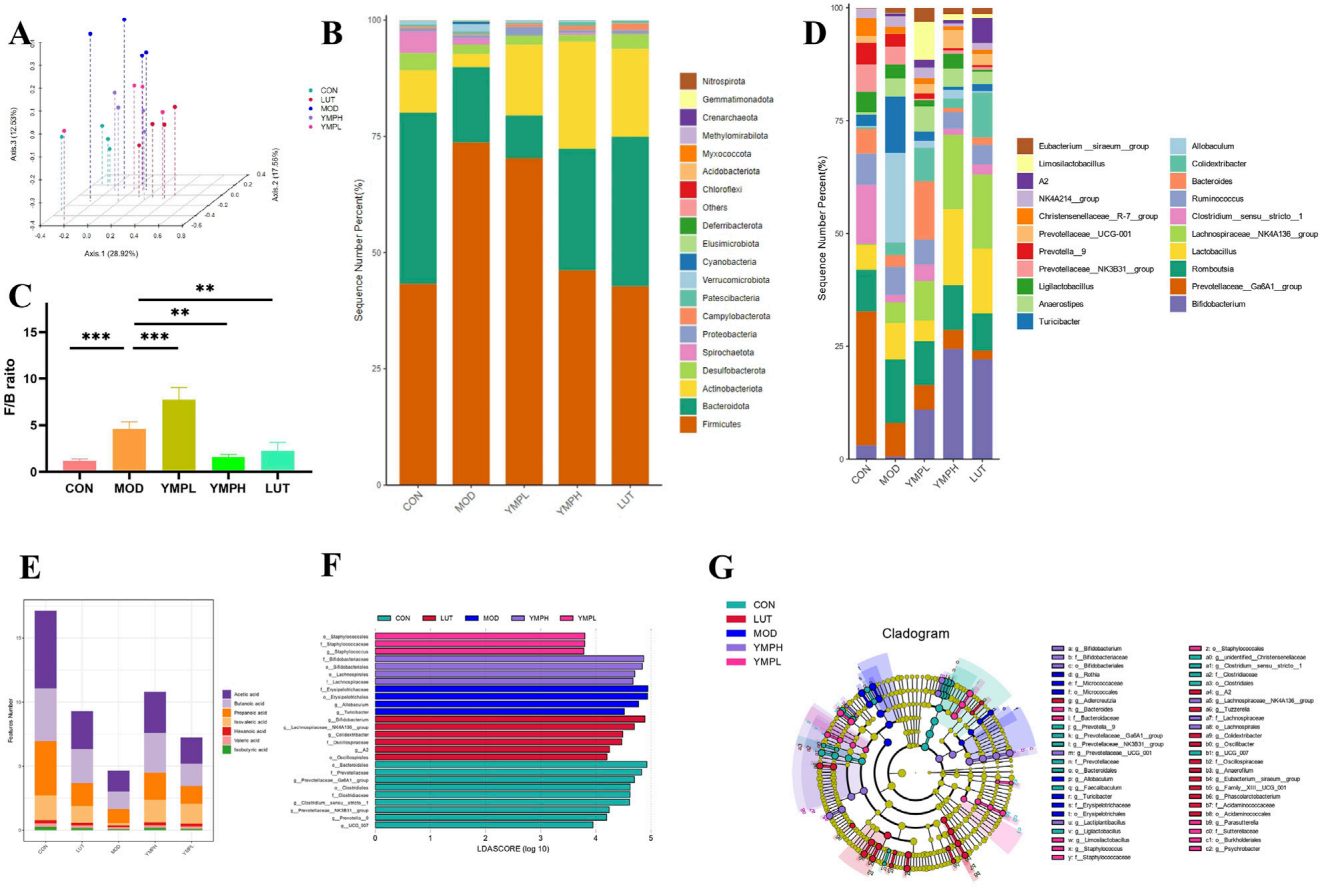
Microbiome composition and microecology following the YMP intervention, including PCoA 3D Diagram based onunweighted Unifrac **(A)**, Average relative abundance of microbiota composition at the phylum level **(B)**, Firmicutes to Bacteroidetes ratio **(C)**, Microbiota composition at the genus level **(D)**, Content of SCFAs **(E)**, LEfSe barplot analysis **(F)**, LEfSe cladogram analysis **(G)**. n = 4.

Furthermore, to evaluate beta diversity, unweighted-uniFrac distance based on PCoA was performed (Figure 5A). There was a significant difference in the intestinal microbiota composition of the mice treated with STZ-HFD and CON groups, indicating that the diabetic intervention had a significant influence and caused dysbacteriosis. These agree with newly published studies on diabetic mice (Huda et al., 2021). The floral diagram showed the relative abundances of common and rare species in each group. The core of the flower showed 671 common OTUs, as shown in Supplementary Figures S2F, and the treatment altered the diversity of the flora. It demonstrated that the improvement of the abnormalities of gut microbiota composition was closely associated with the positive control of YMP.

Determining the phylum and genus level relative abundance of gut microbiota was necessary to determine the composition changed of bacterial community. Five high dominating populations were Firmicutes, Bacteroidota, Actinobacteriota, Desulfobacterota, and Spirochaetota, as Figure 5B illustrates. In particular, the two predominant populations in the gut microbiota are Firmicutes and Bacteroidota. Metabolic disorder is closely correlated with the ratio of Firmicutes to Bacteroidota (F/B) (Magne et al., 2020). YMPH groups enhanced the relative abundance of Firmicutes, and they reversed the increase in Firmicutes generated by STZ and HFD. There were differences in the F/B ratio across all groups (Figure 5C). YMP supplementation was found to enhance *Bifidobacterium*, *Lachnospiraceae_NK4A136_group*, and *Lactobacillus* at the genus level (Figure 5D). In obese mice, *Bifidobacterium* significantly reduced fat content, regulates blood lipids, and corrects aberrant blood sugar levels in addition to controlling weight increase. These findings might be connected to decreased lipid synthesis, improved energy metabolism, exercise, and relief from chronic inflammatory conditions. Increases in *Bifidobacterium* could better regulate the operation of intestinal flora and alter the amounts of bacteria in the body, which may lower the level of inflammatory components (Magne et al., 2020). *Bifidobacterium* promoted the growth of SCFAs-producing, which raised the concentrations of SCFAs in the systemic and fecal environments. This improved dysbiosis and changed the gut microbial ecology. This led to the growth of *Bifidobacterium*-producing bacteria, which in turn raised the amounts of SCFA in the systemic and fecal environments (Li et al., 2020). The microbiome associated with transplantation offers protection against obesity. The SCFAs-producing bacteria *Lachnospiraceae_NK4A136_group*, which was lowered in obese participants, may be partially responsible for these alterations. Notably, gut barrier function is highly linked with changes in the *Lachnospiraceae_NK4A136_group* (Ma et al., 2020). As evidenced by the lower Firmicutes-to-Bacteroidetes ratios and endotoxin-bearing Gram-negative bacteria levels, *lactobacillus* not only reverses HFD-induced gut dysbiosis but also preserves intestinal barrier integrity, lowers metabolic endotoxemia, and inhibits the TLR4/NF-κB signaling pathway. Furthermore, the outcomes of the bacterial functional potential and microbiome phenotypic predictions made demonstrate that *Lactobacillus* treatment enhances the activities of the gut microbiota in relation to immunological response, metabolism, and pathopoiesia (Kang et al., 2022). These organisms have been linked to negative relevance related to FGB and metabolic illnesses associated with diabetes.

In order to identify the representative genus for each group, the LEfSe analysis was carried out, and 26 high-dimensional biomarkers with an LDA score (log_10_) > 3.5 were found. More specifically, the CON group demonstrated high importance and abundant expression of *o_Bacteroidales*, *f_Prevotellaceae*, and *g_Prevotellaceae_Ga6A1_group* (Figure 5F). The cladogram illustrates that all three of these biomarkers are interestingly Bacteroidetes-related at the phylum level, supporting the theory in Figure 5G that *Bacteroidetes* is essential for blood sugar maintenance. Nonetheless, *f_Erysipelotrichaceae* and the genus that corresponded to it shown abundance in MOD. Phylotypes of *Rikenellaceae* which are negatively correlated with obesity were enhanced notably by the intervention of ripened pu-erh tea extract, while *Erysipelotrichaceae* which have positive correlation with obesity were decreased dramatically (Ye et al., 2021). Excitingly, YMP treatment showed quantity privilege of *f_Staphylococcaceae*, *o_Staphylococcales*, *g_Staphylococcus* in YMPL, together with *f_ Bifidobacteriaceae*, *g_Bifidobacterium*, *o_Bacteroidales*, *o_Lachnospirales*, *f_Lactobacillaceae* in YMPH. The result of LUT groups are similar to YMPH groups Blood sugar levels have been shown to be negatively correlated with *Bifidobacteriaceae* and *Lactobacilllaceae*, which can also metabolize inositol to create short chain fatty acids (SCFAs) (Figure 5E) including butyric and propionic acid. These findings pointed to a putative microbial mechanism by which YMP supplementation enhances host health.

### Correlation between SCFA levels and gut microbiota

**FIGURE 6:**
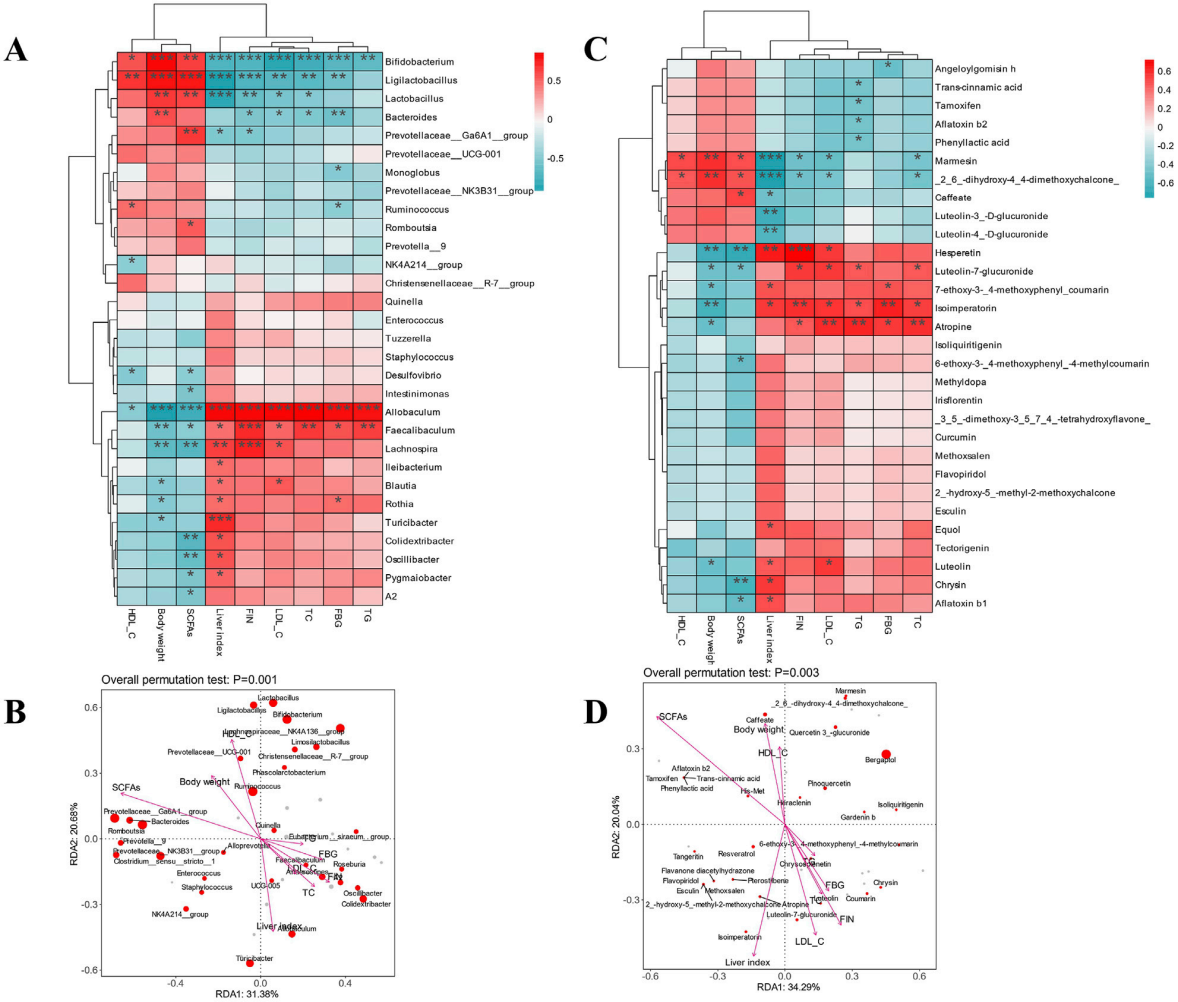
Spearman heatmap analysis between intestinal flora and metabolism index **(A)** and species CCA/RDA ordination chart **(B)**, Spearman heatmap analysis between metabolism index and phenylpropanoid compounds **(C)** and species CCA/RDA ordination chart **(D)**.

Spearman heatmap analysis and canonical correlation analysis/redundancy analysis (CCA/RDA) were used to ascertain the relationship between markers of glycolipid metabolism, SCFAs, serum oxidation indicators, and gut microbiota at the genus level (Figure 6A). HDL-C, body weight, and SCFAs all exhibited a negative connection with other markers. The majority of aberrant serum measures were substantially correlated with *Allobaculum*, *Faecalibaculum*, *Lachnospira*, *Rothia*, and *Turicibacter*. This was consistent with an anti-obesity supplemental polyphenol-rich suppressing the aforementioned many gut bacteria (Chang et al., 2023). There was a favorable correlation between ANGPTL4 expression and *Allobaculum* abundance. ANGPTL4 was a key regulator in lipid metabolism and a circulating medium for gut microbiota and fat deposition. These results offer a theoretical framework for the creation of methods to regulate ANGPTL4 and gut microbiota in order to prevent obesity and associated disorders (Zheng et al., 2021). These flora’s relative abundance offered a great deal of promise as a biomarker for diseases related to glycolipid metabolism. However, the biochemical metrics on hypolipidemic effects showed a high and positive link with *Lactobacillus*, *Bifidobacterium*, *Prevotellaceae_Ga6A1_group*, and *Bacteroides*. Following YMP treatment, there was an increase in the relative abundance of *Bifidobacterium*. This indicated that YMP promoted *Bifidobacterium,* which in turn reduced oxidative stress and reversed levels of TG, LDL-C, and HDL-C. These findings corroborated a prior study’s finding that *Lactobacillus* and *Ligilactobacillus* showed good correlations with serum HDL-C.

CCA/RAD depicted the degree of relationship between biochemical markers and microbiota using the arrow angle, length, and perpendicular distance between microorganisms and parameter variable axes. Other arrows, which are all situated in the fourth quadrant and indicate a negative association consistent with Spearman analysis, are body weight, HDL-C, and total SCFAs. Strong relevance was indicated by the presence of some of the previously reported bacteria with negative significance and close to the arrow in the fourth quadrant. Consequently, controlling the composition of both good and bad bacteria as well as the expression of lipid metabolism proteins may be the interpretation of the mitigating role of YMP. Fecal SCFAs content affects metabolism health and the balance of microecology. Figure 6B demonstrated significant decreases in SCFAs between diabetic and normal mice, suggesting a negative link between metabolic diseases and total SCFAs concentration. In particular, the dominance decreasing components of SCFAs composition in diabetic mice that affected the overall SCFAs content were propionic acid and butyric acid. However, the ingestion of YMP alleviated the side effect. Furthermore, the positive correlation between total SCFAs and *Lactobacillus*, *Bifidobacterium* was in line with findings from earlier research on the roles of SCFAs synthesis.

### Correlation between glycolipid metabolism and phenylpropanoid

The association between serum oxidation indicators, antioxidant SCFAs, phenylpropanoid chemicals, and glycolipid metabolism indicators was investigated using Spearman heatmap analysis and canonical correlation analysis/redundancy analysis (CCA/RDA) (Figures 6C, D). HDL-C, body weight and total SCFAs all exhibited a negative connection with other markers, as would be expected. The majority of aberrant serum parameters were substantially correlated with luteolin and luteolin-7-glucuronide. The relative abundance of luteolin offered a great deal of promise as a biomarker for diseases related to glycolipid metabolism. The arrow angle, length, and perpendicular distance between microorganisms and parameter variable axes were used by CCA/RAD to illustrate the degree of connection between biochemical markers and chemicals. The fourth quadrant contains LDL-C, TC, FIN, and TG, all of which point to a negative association consistent with Spearman analysis. Strong relevance was indicated by the presence of some of the previously reported luteolin and luteolin-7-glucuronide near the arrow in the fourth quadrant, which had negative significance. In HepG2 hepatocytes, luteolin enhanced the expression of key players in cholesterol efflux, including scavenger receptor class B member 1 (SRB1), ATP-binding cassette transporter G1 (ABCG1), and liver X receptor (LXR) α. An inhibitory compound of LXRα reversed the expression of ABCG1 and SRB1 induced by luteolin. In the liver of diet-induced obese mice, luteolin treatment also increased the expression of SRB1, ABCG1, and cholesterol 7 α-hydroxylase (Cyp7α1). In obese mice, luteolin reduced the amount of non-high-density lipoprotein cholesterol and blood cholesterol. Furthermore, luteolin decreased the expression of enzymes linked to gluconeogenesis and improved glucose intolerance in a way that was dependent on LXRα (Park et al., 2020).

### Glycolipid metabolism mechanism via gut microbiome

**FIGURE 7:**
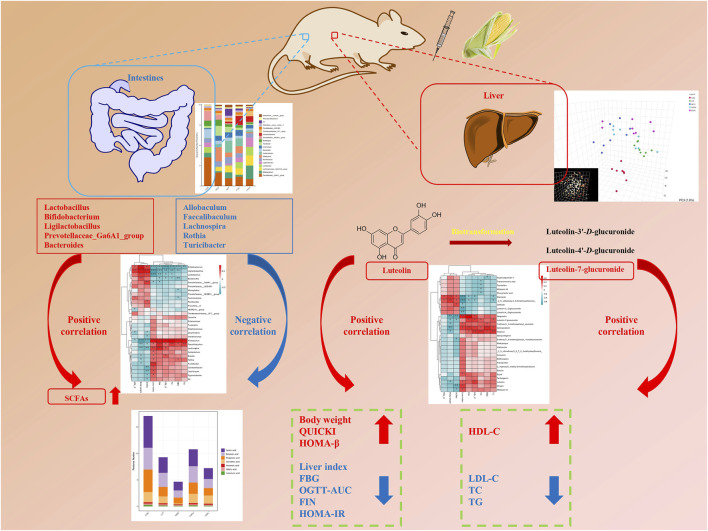
Potential mechanism of improving glucolipid metabolism of YMP via Gut microbiome and Metabonomics.

Phenylpropanoids may be able to improve the variety and structure of the microbiota in order to treat metabolic disorders. First, YMP inhibited α-glucosidase, which led to reduced hydrolysis in the small intestine and increased residual carbohydrate production in the large intestine for the synthesis of SCFAs. There is mounting evidence that luteolin supplementation may have a positive impact on glycolipid metabolism diseases (GLMDs), specifically obesity, diabetes, and insulin resistance (Wang et al., 2021a). Luteolin improved metabolic equilibrium and lessened renal damage by restoring the expression levels of genes linked to Cd-disturbed glycolipid metabolism. Furthermore, through boosting antioxidant capacity and reducing the production of cytokines in the kidney tissues, luteolin showed therapeutic potential against Cd-induced renal oxidative stress and inflammation. Lutein improved the metabolic disruption by attenuating oxidative stress and inflammatory reactions through activating the AMPK/SIRT1/FOXO1 signaling pathway (Wang et al., 2024). Within the luteolin group, *Lactobacillus*, *Bacteroides*, *Roseburia*, and *Butyricicoccus* were the dominating genera. Treatment with luteolin decreased the elevated ratios of *Lactobacillus* and *Prevotella_9* caused by dextran sulfate sodium (DSS). Additionally, KEGG analysis showed that purine metabolism, peptidases, ribosomes, DNA repair and recombination proteins, and pyrimidine metabolism were the primary relationships between gut microbiota (Li et al., 2021). The cross-talk of the gut-brain-liver axis suggests that polyphenols influence the central and enteric neurological systems, as well as the microbial community’s composition, the contents of gut metabolites (SCFAs, bile acids, and endogenous ethanol), and the activation of gut-derived hormones (Wang et al., 2021b). These multielement axes could be understood as strategies of YMP for treating insulin resistance brought on by diabetes, hepatic glycolipid metabolism problems, and dyslipidemia diseases. In conclusion, Figure 7 illustrated the postulated fundamental mechanism through which YMP reduces metabolic disorders.

## Conclusion

Due to their complicated mechanisms of action, multi-component, multi-target, varied pharmacodynamic ingredients, and range of physiological activities, natural products have found widespread application in clinical settings. Following the extraction of the polyphenol enhanced fraction YMP, luteolin and luteolin-7-glucuronide were identified as the principal constituents. *In vivo*, it demonstrated strong hypoglycemic properties. Liver function, abnormal insulin resistance, and liver glycolipid metabolic disorder could all be reversed with a high dosage of YMP (160 mg/kg/d). This work not only described the hypolipidemic activity by reducing hepatic lipid accumulation, but it also provided the first evidence of diabetes associated with metabonomics and microbiota. 16s rRNA provided insight into the gut-mediated mechanism of YMP. YMP may control the composition of the gut microbiota, particularly *Lactobacillus* and *Bifidobacterium*, but more mechanisms require more verification. Without a doubt, the phenylpropanoid-enriched *Zea mays* L. offers a fresh perspective on the supplemental approach to treating metabolic disorders.

## Data Availability

The data presented in the study are deposited in the NCBI BioProject repository, accession number PRJNA1243028.
