# Supplementary material for: Targeted metabolomics unravels the mechanism by phenylpropanoid-rich of the peel of Zea mays L. ameliorates metabolic disorders in diabetic mice through gut microbiota modulation
Source: Front Pharmacol. 2025 Apr 9;16:1551713. doi: 10.3389/fphar.2025.1551713 (PMC12014729; doi:10.3389/fphar.2025.1551713)
Supplement: Supplementary file 1 [file DataSheet1.docx]

Unveiling the mechanism of phenylpropanoid-rich the peel of *Zea mays L.* in ameliorating metabolic disorders in diabetic mice via gut microbiome and metabolomics

Xiaotian Cheng ^a,b,#^, Jinyan He ^a,#^, Yuru Yang ^a^, Yaonan He ^a^, Guangtong Chen ^a^, Bai Ling ^a,b,*^, Andong Wang ^a,*^

^a^ School of Pharmacy, Nantong University, Nantong, Jiangsu 226001, P. R. China

^b^ Department of Pharmacy, The Yancheng Clinical College of Xuzhou Medical University & The First people’s Hospital of Yancheng, Yancheng, Jiangsu 224001, P. R. China

^#^ These authors contributed equally to this work and shared first authorship.

^*^ Corresponding author: Andong Wang ([wangandong19891220@163.com](mailto:wangandong19891220@163.com)), Bai Ling (lingbai@ntu.edu.cn)

**Figure details**


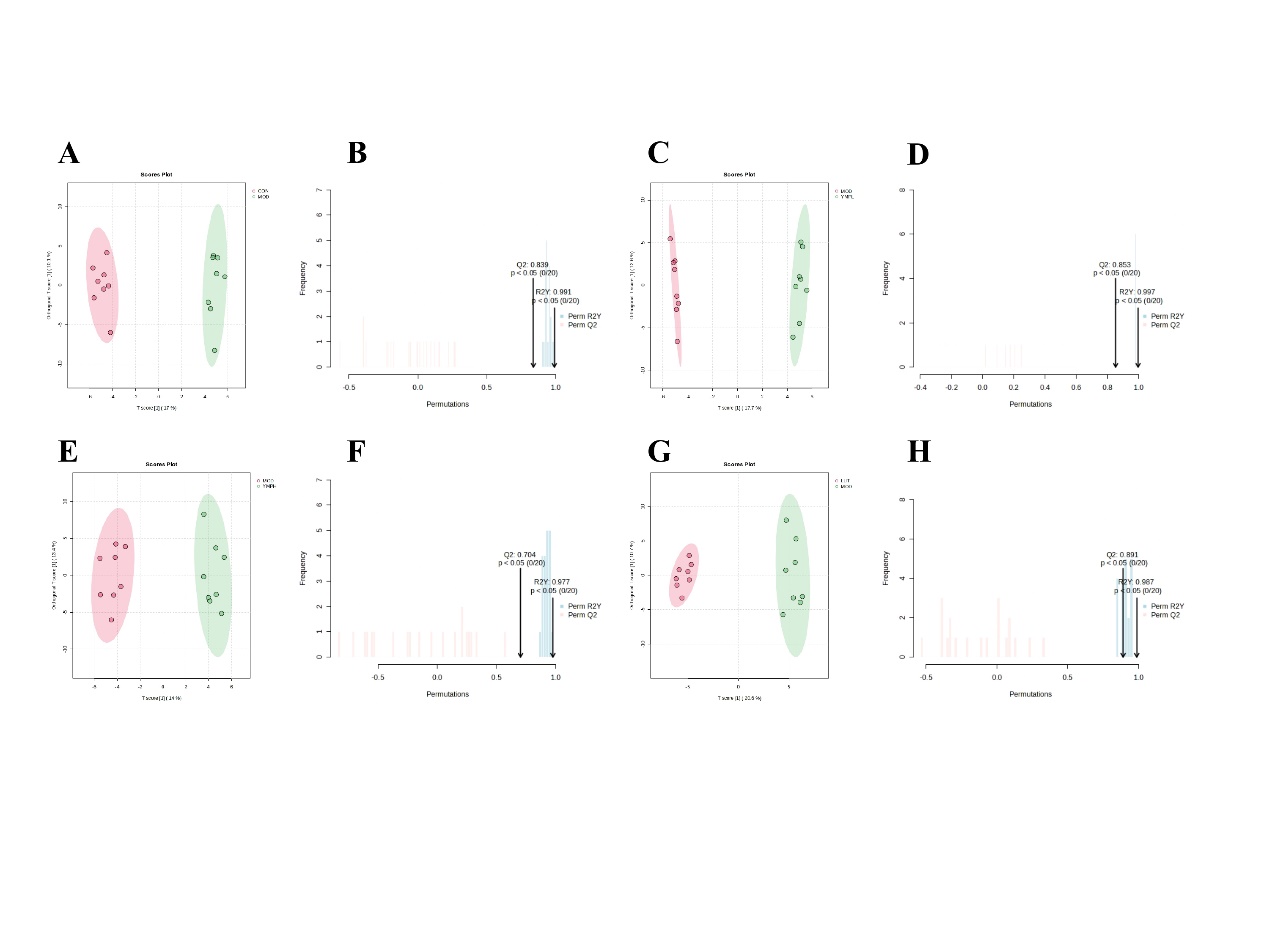


Figure S1 OPLS-DA (CON vs MOD) (A), R2Y and Q2 (CON vs MOD) (B), OPLS-DA (MOD vs YMPL) (C), R2Y and Q2 (MOD vs YMPL) (D), OPLS-DA (MOD vs YMPH) (E), R2Y and Q2 R2Y and Q2 (F), OPLS-DA (MOD vs LUT) (G), R2Y and Q2 (MOD vs LUT) (H). (n=8)


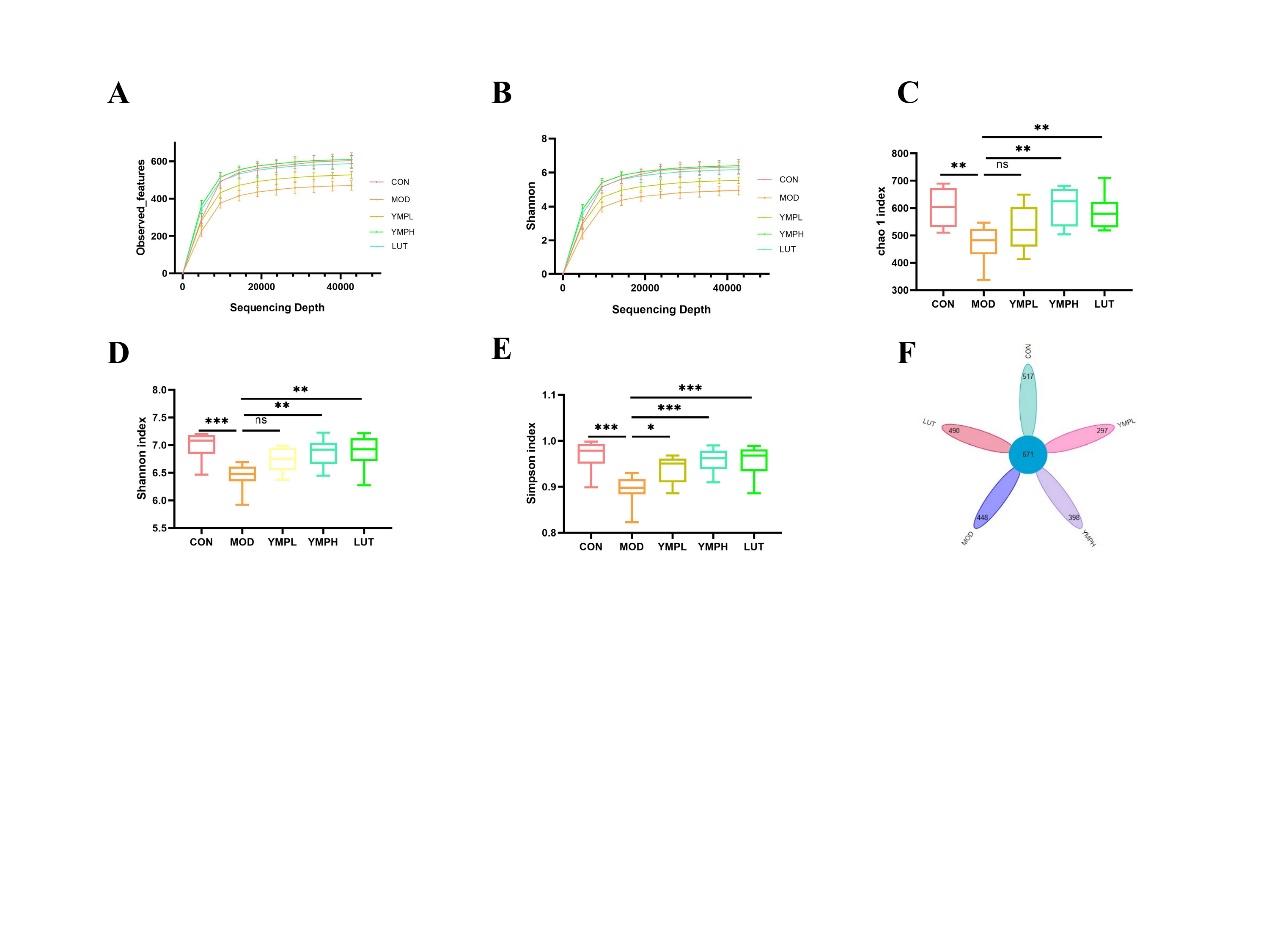


Figure S2 Rarefaction curve (A), Shannon curve (B), Chao1 index (C), Simpson index (D), Shannon index (E), flower diagram of OTUs (F). Note: ^*^ represents for vs MOD group (*p* < 0.05), ^**^ represents for vs MOD group (*p* < 0.01), ^***^ represents for vs MOD group (*p* < 0.001), respectively. n=4
